# Challenges and Strategies for Successful Insulin Pump Therapy in an Elderly Patient With Type 1 Diabetes and Comorbidities: A Case Report

**DOI:** 10.7759/cureus.98093

**Published:** 2025-11-29

**Authors:** Metab Algeffari, Eman A Alotaibi

**Affiliations:** 1 Department of Family and Community Medicine, College of Medicine, Qassim University, Buraydah, SAU

**Keywords:** comorbidity, diabetes mellitus, elderly patients, glycemic control, insulin pump therapy, patient care

## Abstract

Managing type 1 diabetes mellitus (T1DM) in elderly patients with multiple comorbidities presents significant challenges, particularly when complicated by persistent glycemic variability, severe hypoglycemia, and hypoglycemia unawareness. We present a 72-year-old Saudi male with T1DM, ischemic heart disease, hypertension, hypothyroidism, and peripheral neuropathy who was transitioned from multiple daily injections to insulin pump therapy. Prior to intervention, he had frequent severe hypoglycemia and elevated glycated hemoglobin (HbA1c) levels (9.4%, 8.8%). Following the initiation of hybrid closed-loop insulin pump therapy, glycemic control improved (HbA1c 7.7%), and hypoglycemia episodes decreased. However, a language barrier led to a critical insulin dosing error and hospitalization. This case highlights both the benefits and potential risks of insulin pump use in elderly patients. It underscores the importance of addressing language barriers, providing individualized education, and involving caregivers to reduce adverse outcomes. The case adds to the limited data on advanced diabetes technology in the elderly with complex comorbidities. Insulin pump therapy can be effective in elderly patients with T1DM and multiple comorbidities when personalized support and appropriate safeguards are implemented.

## Introduction

The life expectancy of individuals with type 1 diabetes mellitus (T1DM) has significantly increased in recent decades due to advances in diagnostic tools, therapeutic interventions, and patient management strategies. However, as this population ages, the management of T1DM in older adults presents unique challenges, especially in the presence of cognitive decline and various comorbidities [1]. Insulin therapy remains the cornerstone of T1DM treatment, with most patients depending on multiple daily injections (MDI). However, continuous subcutaneous insulin infusion (CSII) therapy using an insulin pump has been shown to provide precise insulin delivery, improved glycemic outcomes, and reduced risk of hypoglycemia, making it an increasingly popular option for patients with T1DM [2-4].

Despite its benefits, CSII therapy requires meticulous management, including the ability to operate the insulin pump and monitor blood glucose levels accurately. These tasks can be particularly challenging for elderly patients with cognitive impairment, reduced manual dexterity, or visual limitations, often necessitating caregiver support [5]. In elderly individuals with T1DM, age-related decline in renal function further contributes to hypoglycemia risk by reducing insulin clearance and impairing renal gluconeogenesis, thereby prolonging insulin action and diminishing glucose recovery capacity [6]. Although the adoption of hybrid closed-loop insulin delivery systems is increasing, real-world evidence regarding their safety, usability, and clinical outcomes in adults over 70 years, particularly those with multiple comorbidities, remains scarce.

The interaction of advanced age and insulin pump use remains a relatively underexplored area. While studies have demonstrated the safety and efficacy of CSII in younger and middle-aged adults with T1DM, evidence regarding its application in older-old or oldest-old populations is limited [5]. Given the increasing prevalence of diabetes and the aging population, understanding the challenges and outcomes of insulin pump therapy in the elderly is critical.

In this case report, we present a rare instance of a 72-year-old patient with T1DM who started on CSII therapy. This case highlights the complexities of managing T1DM in the oldest-old population and underscores the importance of individualized treatment strategies to ensure safe and effective care.

## Case presentation

A 72-year-old Saudi male with T1DM, first diagnosed at the age of 14 years, was transitioned to insulin pump therapy due to persistent glycemic variability, frequent hypoglycemia (three to four episodes per week), and hypoglycemia unawareness while on multiple daily insulin injections (MDIs).

Before pump initiation

The patient was cognitively intact, fully independent in daily living activities, and demonstrated a good understanding of diabetes self-management tasks, including glucose monitoring and insulin administration. His A1C levels were elevated for several years, with fasting glucose levels ranging from 61 to 200 mg/dL and postprandial glucose often exceeding 300 mg/dL and C-peptide level <0.01 ng/mL, confirming long-standing autoimmune diabetes.

His medical history included ischemic heart disease post-acute coronary syndrome in 2022, hypertension, hypothyroidism, and peripheral neuropathy with decreased vibration sensation. Early diabetic nephropathy was indicated by a serum creatinine level of 125.9 μmol/L, an estimated glomerular filtration rate consistent with Stage 3a chronic kidney disease [6], and a mildly elevated albumin-to-creatinine ratio (ACR) (Table 1).

**Table 1:** Laboratory results

Test	Result	Reference range
HbA1c (%)	8.8-9.4	4.6-6.2
Fasting plasma glucose (mg/dL)	61-200	70-130
Postprandial plasma glucose (mg/dL)	>300	140-199
Creatinine (μmol/L)	125.9	53-88
Estimated glomerular filtration rate (eGFR) (mL/min)	53	>60
Albumin-to-creatinine ratio (ACR) (mg/mmol)	1.48	Male: <2.5, Female < 3.5

Despite these comorbidities, he adhered to a low-carbohydrate diet and moderate physical activity, walking 30 minutes three times weekly. His medication regimen included aspirin 81 mg daily, rosuvastatin 20 mg daily, candesartan 16 mg daily, thyroxine 75 mcg daily, insulin degludec 15 IU, and aspart three times before meals. Six months prior to pump initiation, he experienced severe hypoglycemia leading to loss of consciousness and a two-day hospital admission.

**Figure 1:**
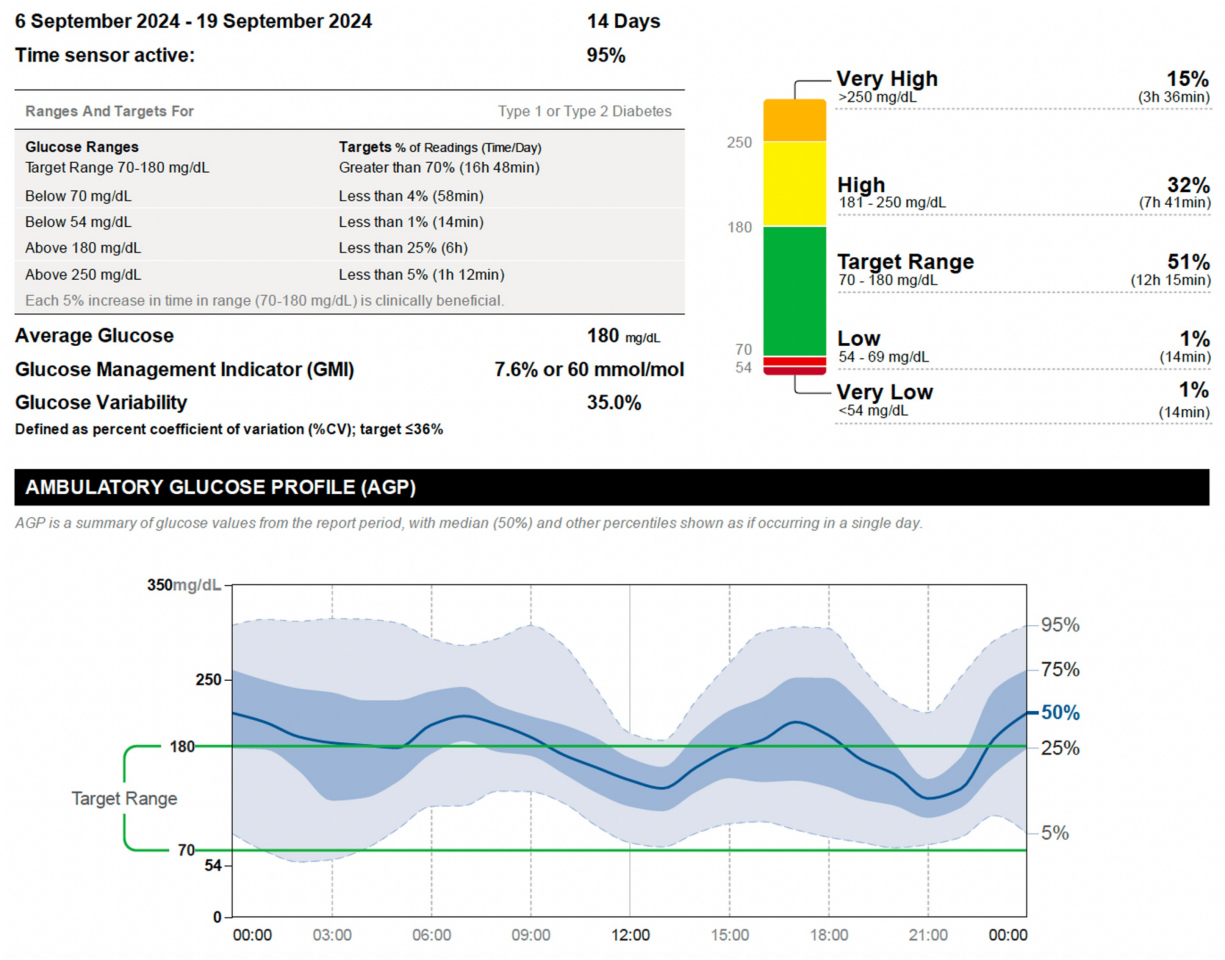
Pre-pump glucose profile showing variability and hypoglycemia Interstitial glucose (IG) data recorded during the two weeks preceding initiation of hybrid closed-loop continuous subcutaneous insulin infusion (CSII). Readings were obtained using the FreeStyle Libre 2 Flash Glucose Monitoring System and uploaded through the LibreView® cloud platform. The profile illustrates marked glycemic variability and recurrent hypoglycemic episodes (<70 mg/dL) prior to transitioning to insulin pump therapy.

He managed glucose variability and adjusted his insulin doses using the FreeStyle Libre 2 Flash glucose monitoring system (CGM) for over four years. During this period, his data showed prolonged periods of hyperglycemia, with some episodes of hypoglycemia. His time above range (TAR), time in range (TIR), and time below range (TBR) were 47%, 51%, and 2%, respectively (Figure 1).

After initiating hybrid closed-loop therapy

**Figure 2:**
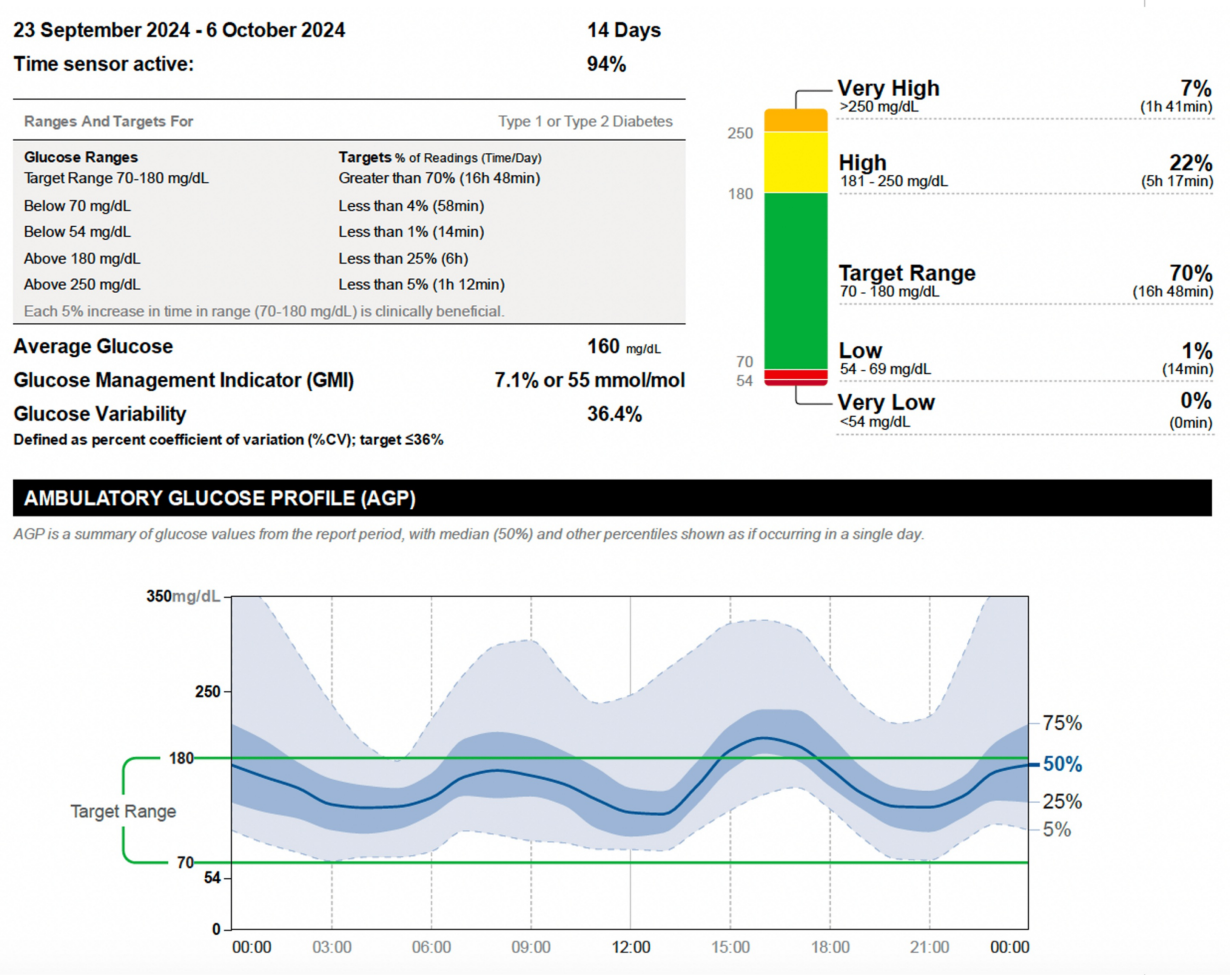
Post-pump glucose profile showing improved stability and reduced hypoglycemia Interstitial glucose (IG) data recorded during the two weeks following initiation of hybrid closed-loop continuous subcutaneous insulin infusion (CSII). Readings were obtained using the FreeStyle Libre 2 Flash Glucose Monitoring System and uploaded through the LibreView® cloud platform. Compared with the pre-pump period, the profile demonstrates improved glycemic stability, increased time-in-range (70–180 mg/dL), and a marked reduction in hypoglycemic episodes.

**Figure 3:**
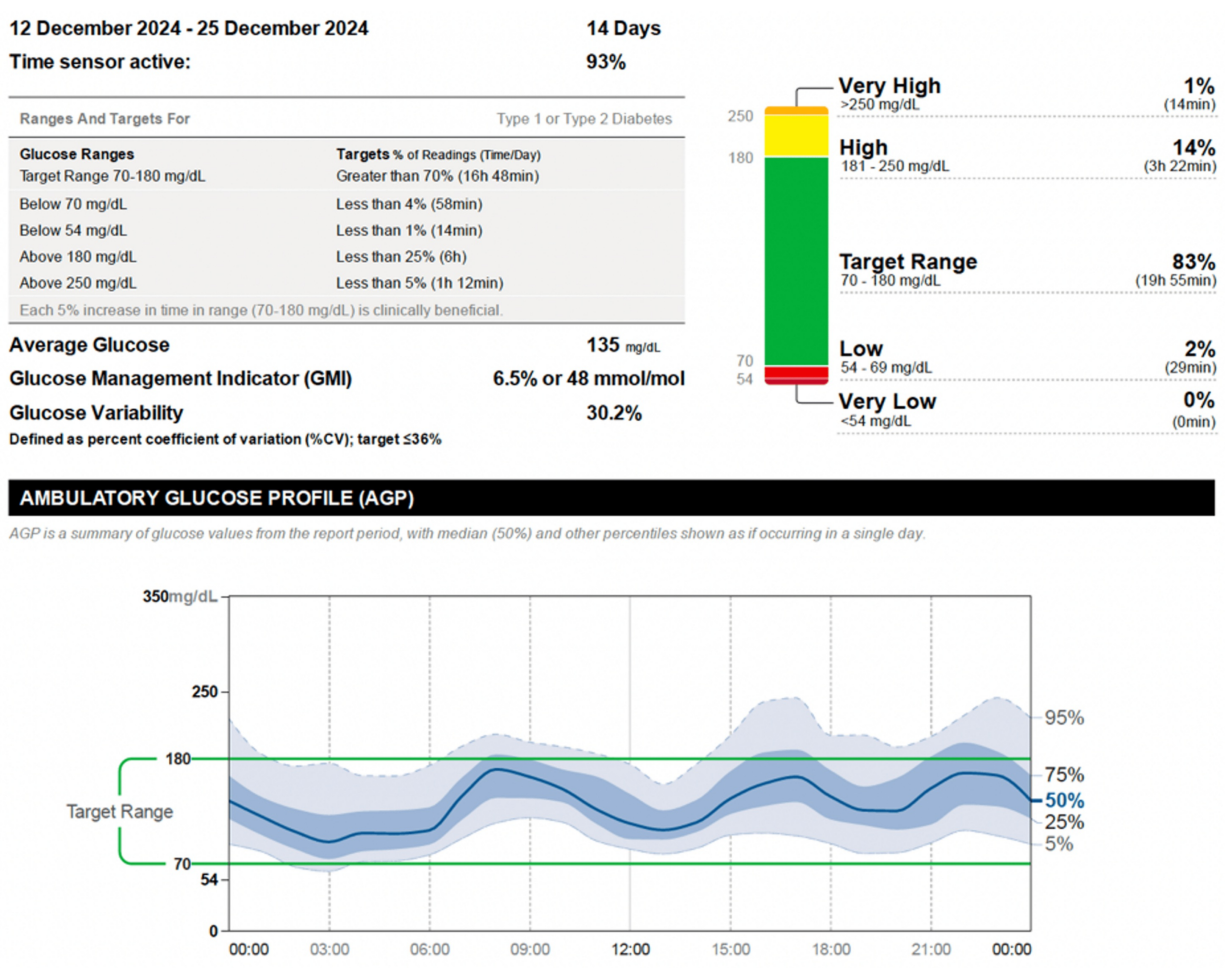
Sustained glycemic control three months after initiating hybrid closed-loop CSII Interstitial glucose (IG) data recorded three months after initiation of hybrid closed-loop continuous subcutaneous insulin infusion (CSII). Readings were obtained using the FreeStyle Libre 2 Flash Glucose Monitoring System and uploaded through the LibreView® cloud platform. The profile demonstrates sustained glycemic stability, high time-in-range (70–180 mg/dL), and minimal hypoglycemic excursions, indicating continued effectiveness and adaptation to the hybrid closed-loop system over time.

The patient was started on a Tandem t:slim X2 insulin pump equipped with the Control-IQ algorithm. Within two weeks, his A1C decreased to 7.7%, and CGM metrics demonstrated substantial improvement, with TAR, TIR, and TBR of 16%, 82%, and 2%, respectively. After three months of therapy, further improvements were noted, with TAR, TIR, and TBR recorded at 15%, 83%, and 2%, alongside a coefficient of variation (CV) of 30.2% (Figures 2-3).

This demonstrates that hybrid closed-loop insulin pump therapy effectively improved blood glucose control, reduced variability, and minimized hypoglycemia episodes over time.

The patient operated the pump independently, had prior experience with carbohydrate counting, and demonstrated proper set changes multiple times. However, due to a language barrier in the pump’s settings guide, during one set change, he connected the tube with the cannula already attached to his body before filling the cannula outside. This error caused the patient to receive a dose of insulin, leading to severe hypoglycemia that required hospital intervention. The healthcare team removed the pump, and the patient was temporarily transitioned back to insulin pens until stabilization.

Actions included advocating for updating the pump system to include the patient’s primary language, emphasizing caregiver involvement during set changes, and providing additional training. This case highlights the benefits of insulin pump therapy in elderly patients with T1DM and multiple comorbidities, demonstrating its potential to enhance glycemic control, reduce hypoglycemia, and improve overall quality of life. It also emphasizes the importance of addressing language barriers and ensuring caregiver support to maximize safety and efficacy.

## Discussion

The management of T1DM in elderly patients with multiple comorbidities, as highlighted in this case, presents unique challenges. Advanced diabetes technologies, including CSII, have shown significant promise, particularly in addressing glycemic variability and hypoglycemia unawareness in older adults. Below, we contextualize the outcomes of this case with other documented rare instances of insulin pump therapy in similar populations. A case report by McGregor et al. [7] described a 72-year-old patient undergoing total pancreatectomy for multifocal pancreatic cancer who achieved long-term glycemic stability using an insulin pump, with an HbA1c reduction to 7.0% three years post operation. This highlights the role of CSII in stabilizing blood glucose even in extreme cases, such as pancreatic diabetes [7]. Moreover, Cook et al. [8] discussed the use of insulin pumps in geriatric populations and emphasized that, while advanced age introduces challenges such as cognitive decline and physical impairments, with adequate caregiver training and patient support, CSII therapy can safely and effectively reduce hypoglycemic events and hospitalizations [8]. These findings align closely with our case, where glycemic control improved substantially post-CSII, reducing hypoglycemic episodes and enhancing quality of life. In our patient, A1C improved from 9.4% to 7.7% following initiation of hybrid closed-loop CSII, representing a clinically meaningful reduction associated with a lower risk of microvascular complications such as retinopathy and nephropathy. Continuous glucose monitoring data also demonstrated a coefficient of variation (CV) of 30.2%, reflecting moderate glycemic stability. This degree of variability is within the recommended threshold (<36%) and indicates improved glucose consistency, although further optimization could enhance stability and minimize hypoglycemic risk. Allen et al. [9] detailed the successful use of insulin pumps in elderly patients with dementia in assisted living facilities. Even among cognitively impaired patients, insulin pumps reduced glycemic variability and hypoglycemia when supported by trained caregivers. While our patient operated the pump independently, this underscores the importance of personalized training and the integration of caregiver involvement when necessary [9].

In this case, a significant barrier was the language mismatch in the pump’s instructional materials, leading to a severe dosing error. The diabetes and aging study of Moffet et al. highlights that elderly patients with diabetes are at increased risk of severe hypoglycemia and subsequent falls, which can have severe consequences [10]. This supports the need for clear, comprehensible instructions in the patient’s primary language to minimize errors and associated risks. The dangers of severe hypoglycemia in elderly diabetic patients are well-documented. McCoy et al. demonstrated that patients reporting severe hypoglycemia face increased mortality risks, emphasizing the need for preventive strategies [11]. Additionally, the ACCORD study by Bonds et al. found a significant association between symptomatic hypoglycemia and mortality in individuals with diabetes [12]. These findings are crucial in understanding the implications of hypoglycemia in this case, where language barriers contributed to a dosing error that led to severe hypoglycemia requiring hospitalization. Furthermore, Whitmer et al. highlighted that recurrent hypoglycemic episodes in older patients with diabetes are associated with an increased risk of dementia [13]. Lacy et al. further supported this, noting that older adults with diabetes, including T1DM, are at risk for cognitive decline when compared to those without diabetes [14]. These insights emphasize the importance of preventing hypoglycemic events through tailored interventions, as demonstrated in this case with the initial success of CSII.

A retrospective study conducted in Bogota, Colombia, evaluated the efficacy and safety of sensor-augmented insulin pump therapy with a low-glucose suspend feature in elderly patients. The study included individuals aged over 60 who had been using the pump for more than one year. The findings showed significant reductions in hypoglycemia and improved glycemic control, with an average A1C of 7.4% after prolonged pump use [15]. However, unlike the presented case, the Bogota study involved patients who initiated pump therapy at a younger age, allowing for prolonged adaptation and familiarity with the technology. In contrast, initiating insulin pump therapy in older adults, as seen in this case, introduces unique challenges such as language barriers, manual dexterity issues, and comorbidities. These differences highlight the necessity of individualized approaches and tailored interventions when introducing advanced technologies to elderly populations [15]. Hybrid closed-loop systems offer substantial advantages for older adults with T1DM, including reduced glycemic variability and fewer hypoglycemic episodes [16]. Nevertheless, these technologies also demand cognitive engagement, preserved manual dexterity, adequate visual acuity, and financial feasibility [17]. The language interface issue observed here further emphasizes the importance of user-centered and culturally adapted human-factors design to ensure device safety and usability [17]. Additionally, policies have also been studied, emphasizing the role of systemic support in facilitating access to advanced diabetes technologies for elderly populations. These policies significantly increased the utilization of insulin pumps among older adults, improving glycemic control and reducing diabetes-related complications [18]. As this report represents a single case, the findings should be interpreted with caution; however, they align with emerging evidence from comparative studies supporting the feasibility and safety of hybrid closed-loop systems in older adults. These findings further reinforce the importance of structured education and personalized interventions in maximizing the benefits of CSII therapy. Equally important, the integration of psychoeducational support and ongoing emotional engagement empowers elderly patients to take an active role in their diabetes management, promoting confidence, adherence, and sustained therapeutic success.

## Conclusions

This case highlights the potential benefits of CSII in achieving better glycemic control for elderly patients with complex medical conditions. The reduced glycemic variability and enhanced stability observed in these cases highlight the transformative potential of insulin pump therapy. However, addressing barriers such as language limitations, caregiver support, and tailored education is crucial for optimizing outcomes. These findings contribute to the growing evidence base, demonstrating that, with appropriate interventions, insulin pump therapy can significantly improve the quality of life for elderly patients with T1DM.
